# P-1979. Impact of an Electronic Health Record Transition on Penicillin Allergy Documentation in a Pediatric Research Hospital

**DOI:** 10.1093/ofid/ofaf695.2146

**Published:** 2026-01-11

**Authors:** Savanna Brinkman, Kristen Hughes, Delia Allen, Elisabeth Adderson, Patricia J Barker

**Affiliations:** St. Elizabeth Healthcare, Edgewood, KY; St. Jude Children’s Research Hospital, Memphis, Tennessee; St. Jude Children’s Research Hospital, Memphis, Tennessee; St. Jude Children's Research Hospital, Memphis, Tennessee; St. Jude Children's Research Hospital, Memphis, Tennessee

## Abstract

**Background:**

Accurate allergy documentation is crucial for preventing adverse drug reactions and optimizing antimicrobial stewardship. Electronic health record (EHR) transitions pose challenges to maintaining consistent allergy labeling, especially in populations where allergy de-labeling efforts have occurred. Interoperability between healthcare systems can further complicate allergy reconciliation if documentation is inconsistent. This project is a quality improvement initiative to quantify and verify allergy labeling discrepancies after an EHR transition in penicillin allergic patients.

**Figure:**
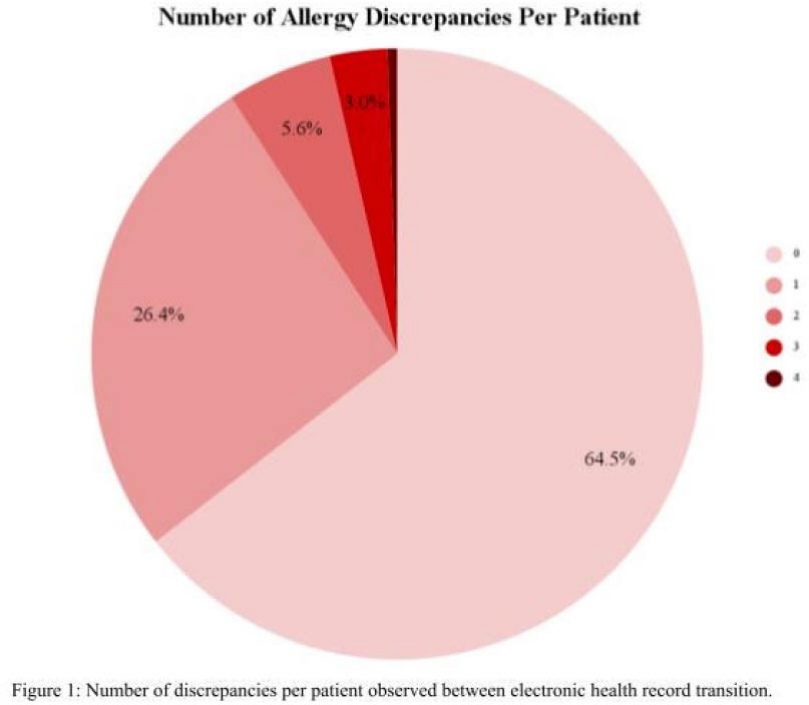

**Figure:**
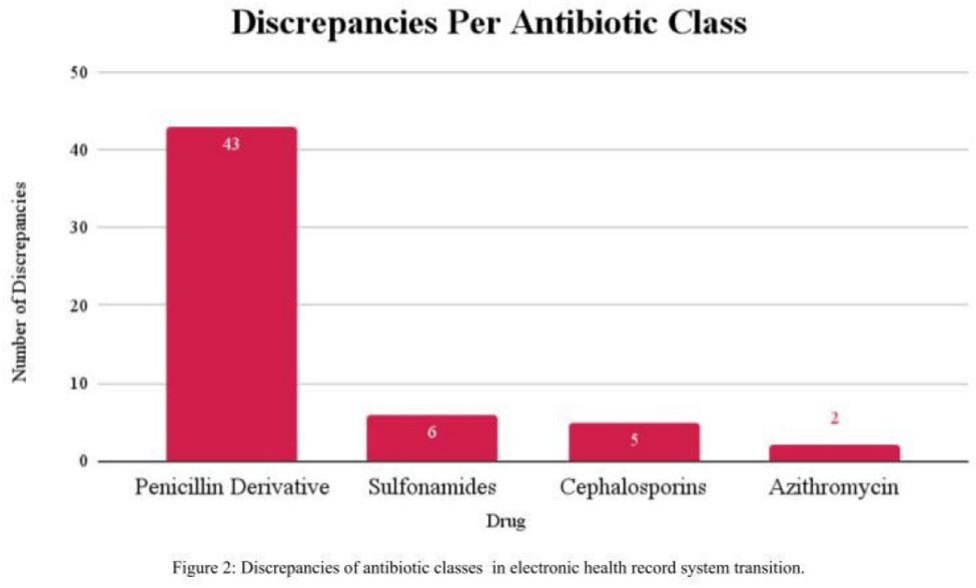

**Methods:**

A retrospective chart review was conducted of 197 patients with active penicillin or penicillin derivative allergies listed in the institution’s EHR, one year following the transition between commercial EHRs. Allergy lists were compared between systems to identify medication allergy discrepancies. If the allergy occurred after EHR transition it was not included as a discrepancy. Measures included: the number of allergy label changes per patient, rate of inappropriate relabeling of previously de-labeled allergies, and inappropriate removal of previously confirmed allergies. Messages were sent to active patients for verification purposes, and the EHR was revised accordingly.

**Results:**

A total of 87 medication allergy discrepancies were identified in 72/197 (36.5%) patients. Antibiotics were 56 (64.3%) of the identified discrepancies with penicillin or penicillin derivatives being the most common antibiotic 43/56 (76.8%). Three cases of inappropriate penicillin allergy relabeling occurred despite previous de-labeling efforts, three patients were inappropriately labelled as not penicillin allergic, and one patient was relabeled with sulfa allergy. Patient messages were sent to 37/62 (59.7%) active patients with 18/37 (48.6%) response. Allergies were patient reported as correct in 17/18 (94.4%) suggesting an incomplete legacy system list.

**Conclusion:**

EHR transitions significantly impact allergy documentation with over one-third of patients in this study experiencing discrepancies. Our findings indicate a need for standardized cross-facility allergy reconciliation processes to prevent documentation errors and improve safety.

**Disclosures:**

Elisabeth Adderson, MD, Shionoji Inc.: Advisor/Consultant

